# Evolutionary and sequence-based relationships in bacterial AdoMet-dependent non-coding RNA methyltransferases

**DOI:** 10.1186/1756-0500-7-440

**Published:** 2014-07-10

**Authors:** Jeanneth Mosquera-Rendón, Sonia Cárdenas-Brito, Juan D Pineda, Mauricio Corredor, Alfonso Benítez-Páez

**Affiliations:** 1 Bioinformatics Analysis Group – GABi, Centro de Investigación y Desarrollo en Biotecnología – CIDBIO, 111221 Bogotá, D.C, Colombia; 2 GEBIOMIC Group, Faculty of Life Sciences, Universidad de Antioquia, Medellín, Colombia; 3 APOLO Computing Center. EAFIT, Medellín, Colombia

**Keywords:** Molecular evolution, RNA methyltransferases, Bacteria, Conserved sequence motifs, Antibiotic resistance

## Abstract

**Background:**

RNA post-transcriptional modification is an exciting field of research that has evidenced this editing process as a sophisticated epigenetic mechanism to fine tune the ribosome function and to control gene expression. Although tRNA modifications seem to be more relevant for the ribosome function and cell physiology as a whole, some rRNA modifications have also been seen to play pivotal roles, essentially those located in central ribosome regions. RNA methylation at nucleobases and ribose moieties of nucleotides appear to frequently modulate its chemistry and structure. RNA methyltransferases comprise a superfamily of highly specialized enzymes that accomplish a wide variety of modifications. These enzymes exhibit a poor degree of sequence similarity in spite of using a common reaction cofactor and modifying the same substrate type.

**Results:**

Relationships and lineages of RNA methyltransferases have been extensively discussed, but no consensus has been reached. To shed light on this topic, we performed amino acid and codon-based sequence analyses to determine phylogenetic relationships and molecular evolution. We found that most Class I RNA MTases are evolutionarily related to protein and cofactor/vitamin biosynthesis methyltransferases. Additionally, we found that at least nine lineages explain the diversity of RNA MTases. We evidenced that RNA methyltransferases have high content of polar and positively charged amino acid, which coincides with the electrochemistry of their substrates.

**Conclusions:**

After studying almost 12,000 bacterial genomes and 2,000 patho-pangenomes, we revealed that molecular evolution of Class I methyltransferases matches the different rates of synonymous and non-synonymous substitutions along the coding region. Consequently, evolution on Class I methyltransferases selects against amino acid changes affecting the structure conformation.

## Background

Post-transcriptional modifications of nucleotides in RNA molecules, such as ribosome and transfer RNA (rRNA and tRNA, respectively), is a process observed in the three major kingdoms of life: Archea, Eukarya and Bacteria. It is evidenced as a sophisticated epigenetic mechanism involved in translation accuracy and gene expression control. RNA modifications appear to confer structural stability [1,2] and to participate in translation fidelity [3,4]. Among the wide variety of modifications found in rRNAs and tRNAs, uridine isomerization (pseudourdine synthesis) and the methylation of nucleobases and/or ribose moieties of nucleotides are predominantly present in these central biomolecules [5,6]. Bacterial RNA modification is enzyme-dependent, thus involving a broad variety of protein families that are highly specialized in both the reaction and substrate to be modified [5,6]. Notwithstanding, very recent reports have demonstrated dual specificity and activity [7-10]. One interesting group of proteins acting as RNA-modifying enzymes is composed of AdoMet- (or S-adenosyl-L-methionine) dependent RNA methyltransferases (MTases). Globally, enzymes that methylate RNA comprise two major classes of MTases according to their structure core: i) Rossmann-Fold MTases (RFM) including almost all the N and C methylases and modify nucleobases; ii) SPOUT MTases consisting of 2’-*O*-methylases which act essentially in tRNAs with very few exceptions [11,12]. However, a later classification of MTases distinguishes five structurally different classes of MTases denoted as I (RFM), II, III, IV (SPOUT) and V [13]. Interestingly, the global sequence conservation among all the MTases classes is poor, which hinders the proposal of phylogenetic relationships. However, they structurally manifest an analogous architecture as a result of using AdoMet as a cofactor of the methyltransfer reaction [13,14]. Class I MTases comprise most rRNA-modifying enzymes (and DNA MTases) showing a fair degree of sequence similarity [6]. The low degree of sequence similarity observed in the predominantly Class I MTases hinders study of their evolutionary history. Although an extensive duplication and specialization process in evolution is thought to produce multiple families of known RNA MTases, the possibility that multiple lineages of RNA MTases can emerge cannot be ruled out [12,13]. Most rRNA MTases are well-conserved only in bacteria and cannot be traced in other kingdoms such as Eukarya. Nevertheless, a few genes (i.e., *rsmA*) display a wide phylogenetic distribution that confers these conserved MTases a relevant role in decoding both the function and biogenesis of the ribosome [12,15,16]. Certain indigenous RNA methylations have been characterized as being pivotal to maintain ribosome fidelity [7,17-20], and one of them has even been characterized as indispensable for cell growth, indicating a critical role for the proper ribosome function [21]. Alternatively, the mutations at ribosome genes, such as rRNA MTases, appear to be frequently associated with conferring antibiotic resistance. One of the first reported cases is *rsmA* mutation inactivating the RsmA function and promoting Kasugamycin resistance [22-24]. Another well-known case of antibiotic resistance associated with mutations in rRNA MTases is the *rsmG* gene [25-27]. The global mechanisms of the initial state of low-level resistance and the later acquisition of high-level resistance seems similar among strains and genes [27,28]. All the above-mentioned effects of RNA methylation deficiency on cell physiology, as well as the well-known antibiotic resistance phenomena by plasmid-encoded RNA MTases [29-33], are thought to design new antimicrobial strategies.

With the recent characterization of YhiR as the RlmJ MTase that acts on 23S rRNA from *Escherichia coli*[34], the full set of RNA MTases for this model organism have been depicted (see Table 1). Currently, research aims are conducted to disclose both the RNA modifications and responsible enzymes in other model organisms as part of the Modomics field of RNA biology. Notwithstanding, further sequence, structural, and functional characterizations of the known RNA MTases are absolutely essential to: i) clarify the critical amino acids for the function and specificity of MTases; ii) disclose potential new catalytic mechanisms; iii) study the structural rearrangements that some MTases undergo to perform their functions; iv) acquire knowledge of the dual activities of RNA MTases, which are becoming a more frequent event than expected; and v) shed light on the evolutionary origin and relationships among RNA MTases. In recent years, several three-dimensional structures have been solved and some offer insights into catalytic mechanisms of nucleotide methylation [8,35-37]. Similarly, relevant genomic studies have presented important phylogenetic and evolutionary features of RNA MTases [11,38]. Moreover, with the full set of known RNA MTases characterized for the model organism *Escherichia coli*, new large-scale sequence and genomic studies into the function, variation and diversity of these enzymes responsible for RNA methylation can lead to a better understanding of the origin of this superfamily of enzymes and shed light on both their evolutionarily meaning as well as the link between RNA methylations and bacterial antibiotic resistance.

**Table 1:** **Set of the *E. coli *** **RNA MTases used as bait in this study**

**Name**	**Alternative Name(s)**	**Substrate**	**Modification**	**MTase Family**^**A**^	**Uniprot**	**Reference**
RlmA	RrmA, YebH	23S rRNA	m^1^G745	Class I (RFM)	P36999	[39]
RlmB	YfjH	23S rRNA	Gm2251	Class IV (SPOUT)	P63177	[40]
RlmC	RumB, YbjF	23S rRNA	m^5^U747	Class I (RFM)	P75817	[41]
RlmD	RumA, YgcA	23S rRNA	m^5^U1939	Class I (RMF)	P55135	[42]
RlmE	FtsJ, MrsF, RrmJ	23S rRNA	Um2552	Class IV (SPOUT)	P0C0R7	[43]
RlmF	YbiN	23S rRNA	m^6^A1618	Class I (RFM)	P75782	[44]
RlmG	YgjO	23S rRNA	m^2^G1835	Class I (RFM)	P42596	[45]
RlmH	YbeA	23S rRNA	m^3^Ψ1915	Class IV (SPOUT)	P0A8I8	[44]
RlmI	YccW	23S rRNA	m^5^C1962	Class I (RFM)	P75876	[46]
RlmJ	YhiR	23S rRNA	m^6^A2030	Class I (RFM)	P37634	[34]
RlmK/L*	YcbY	23S rRNA	m^7^G2069 / m^2^G2445	Class I (RFM)	P75864	[47-49]
RlmM	YgdE	23S rRNA	Cm2498	Class I (RFM)	P0ADR6	[50]
RlmN	YfgB	23S rRNA / tRNAs	m2A2503 / m2A37	Radical SAM	P36979	[7,51]
RsmA	KsgA	16S rRNA	m^6^_2_A1518 / m^6^_2_A1519	Class I (RFM)	P06992	[52]
RsmB	RrmB, YhdB, Sun	16S rRNA	m^5^C967	Class I (RFM)	P36929	[53,54]
RsmC	YjjT	16S rRNA	m^2^G1207	Class I (RFM)	P39406	[55]
RsmD	YhhF	16S rRNA	m^2^G966	Class I (RFM)	P0ADX9	[56]
RsmE	YggJ	16S rRNA	m^3^U1498	Class IV (SPOUT)	P0AGL7	[57]
RsmF	YebU	16S rRNA	m^5^C1407	Class I (RFM)	P76273	[58]
RsmG	GidB	16S rRNA	m^7^G527	Class I (RFM)	P0A6U5	[27]
RsmH	MraW, YabC	16S rRNA	m^4^C1402	Class I (RFM)	P60390	[18]
RsmI	YraL	16S rRNA	Cm1402	Class I (RFM)	P67087	[18]
RsmJ	YhiQ	16S rRNA	m^2^G1516	Class I (RFM)	P68567	[59]
TrmA	RumT	tRNAs / tmRNAs	m^5^U54 / m^5^U341	Class I (RFM)	P23003	[9,60]
TrmB	YggH	tRNAs	m^7^G46	Class I (RFM)	P0A8I5	[61]
TrmD	TrmD	tRNAs	m^1^G37	Class IV (SPOUT )	P0A873	[62]
TrmH	SpoU	tRNAs	Gm18	Class IV (SPOUT )	P0AGJ2	[63]
TrmJ	YfhQ	tRNAs	Um32 / Cm32	Class IV (SPOUT)	P0AE01	[64]
TrmL	YibK	tRNAs	Um34 / Cm34	Class IV (SPOUT)	P0AGJ7	[65]
TrmN6	YfiC	tRNAs	m^6^A37	Class I (RFM)	P31825	[66]
MnmC^B^	YfcK, TrmC	tRNAs	mnm^5^U	Class I (RFM)	P77182	[67,68]
CmoA	YecO	tRNAs	cmo^5^U	Class I (RFM)	P76290	[69]
CmoB	YecP	tRNAs	cmo^5^U	Class I (RFM)	P76291	[69]

*We split this fused-domain MTase to assume independent MTases during the homolog search. A) Classification according to amino acid motifs and published structure when available; *RFM* = Rossmann Fold Methyltransferase; *SPOUT* = Similar to the SpoU MTase structure. B) Only refers to the N-terminal MTase domain of the bi-functional MnmC protein.

## Results and discussion

### Evolutionary conservation of RNA MTases

**Figure 1:**
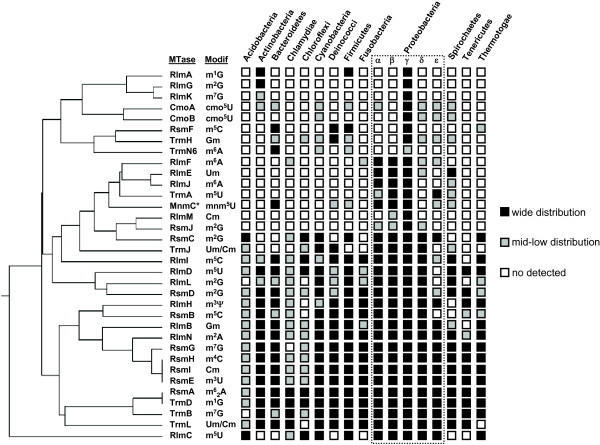
**Phylogenetic distribution of the RNA MTases across bacteria phyla.** The UPGMA dendrogram of the RNA MTases (Table 1) is shown according to their distribution in major bacterial phyla. A presence/absence pattern was categorized as follows: wide distribution (black-filled squares), where the respective gene is present in almost all the phyla species; mid-low distribution (gray-filled squares), where the respective gene is present in ~50% of the phyla species; and undetected (white-filled squares), where the respective gene showed no clear homologs.

**Figure 2:**
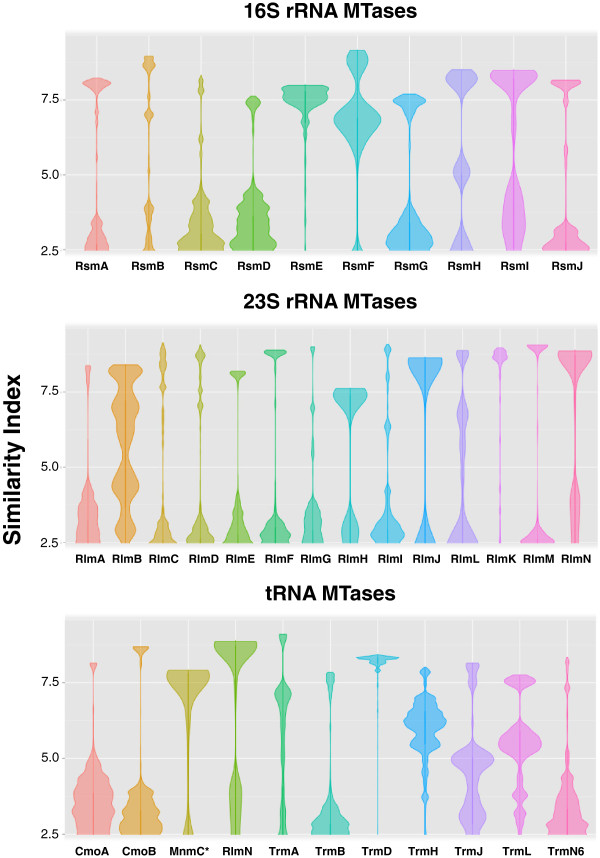
**Distribution of the RNA MTase sequence motifs across bacterial genomes.** Using the amino acid profiles inferred from the probabilistic methods, a search for the proteins matching the RNA MTase sequences was done. Information on query and alignment length, and on the score for amino acid replacements was used to draw the density violin plots per family and class of RNA MTases. Orthology was considered for those hits (with few exceptions) with a Similarity Index higher than 7.5, whereas paralogy was considered for hits with a Similarity Index 5.0 to 7.5. *Refers to the N-terminal domain of the bi-functional MnmC enzyme.

After collecting the full set of RNA MTases acting in both rRNAs and tRNAs from *Escherichia coli* (see Table 1), we recovered homologs for each family of RNA MTases by using this set of proteins as a query in a Blastp-based searching. Thus, we recovered almost 3,000 different sequences, which represent a high level of diversity for these MTases in Eubacteria. We built a UPGMA-based dendrogram using the phylogenetic information obtained from RNA MTases across bacterial species (Figure 1). This dendrogram reflects relationships among RNA MTase families according to their distribution in major bacteria phyla. Globally, two major groups of MTases are observed, considered to be those enzymes with a mid to low conservation across species and those that are very well-conserved. In this latter group, a core of enzymes required for the proper ribosome function is distinguished. Consequently, 16S rRNA MTases RsmG, RsmH/I, and RsmE emerge as the highly conserved accessory proteins of the prokaryote ribosome, and their relevance for translation is further supported by the fact that their products m^7^G527, m^4^Cm1402 and m^3^U1498, respectively, lie on rRNA regions and play pivotal roles in the decoding function [70,71]. Likewise, the high evolutionary conservation of RsmB (responsible for the m^5^C967 modification) matches an important role of its target in the ribosome function [72]. Regarding the evolutionary conserved pattern of 23S rRNA MTases, we observed that RlmH, RlmB and RlmN, producing m^3^Ψ1915, Gm2251 and m^2^A2503, respectively, are present in most bacterial species with very few exceptions. Similarly to the conserved 16S rRNA MTases, these three enzymes act on central sites for the ribosome function located close to the Petidyl Transferase Center (PTC). The pivotal role of these modifications is well supported given that inactivation of respective 23S rRNA MTases function has been shown to have negative effects on translation and cell physiology [7,51,73,74]. Interestingly, almost all of these sites and/or regions of 16S and 23S rRNA showing a conserved methylation pattern appear to be associated with antibiotic resistance. As a consequence, alteration of methylation patterns on rRNA has been associated aminoglycoside [24,27], tetracycline [75], tylosin [76], linezolid [77,78], and chloramphenicol resistance [79] as well as PhLOPSA multiresistance [80]. All this evidence could indicate that rRNA methylation emerges as a new molecular mechanism mediating bacterial resistance. This last issue is intriguing given that mutations in RNA MTases often produce associated fitness cost [18,44,51]. Consequently, the process of antibiotic resistance acquisition, normally initiated with low-level resistances, requires further study in order to disclose the genetic and physiology basis of this short-term evolutionary process. Regarding the RNA MTases acting on tRNAs, the TrmD, TrmB, and TrmL MTases also appear to be highly conserved among bacteria. TrmD and TrmL are responsible for the pivotal modifications occurring in the anticodon region of tRNAs, where they are directly involved in proper mRNA decoding [19,65]. In global terms, approximately half the studied enzymes can constitute the minimal set of methylations at rRNA and tRNAs required for life. This estimation slightly differs when allelic genes encoding enzymes responsible of universally conserved modifications like m^5^U54 are considered [81]. In a similar manner, we hypothesize that the modification performed by the poorly conserved RsmF protein can be made by other enzymes because several paralogs of this protein have been detected (see Figure 2). Strikingly, the RNA MTases responsible for m^6^A modifications seem to display low conservation (except the RsmA dimethylase enzyme). This fact could indicate that acquisition of this modification type is a recent event during evolution.

### Sequence-based relationships among RNA MTases (the similarity network)

**Figure 3:**
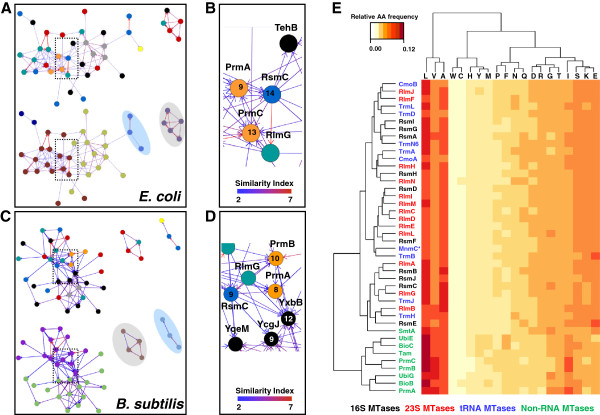
**Sequence motifs and amino acid content-based MTase clustering.** A similarity network approach to distinguish the sequence relationships among the RNA MTases. Edges are represented by Similarity Index scores (see Amino acid profiles at Methods) and Nodes are denoted by a function assigned to each MTase as follows: 16S rRNA MTases (blue), 23S RNA MTases (green), tRNA MTases (red), ribosome protein MTase (orange), cofactor/vitamin biosynthesis MTase (gray), unknown function (black). The numbers located inside the nodes in **B** and **D** panels indicate connectivity (number of interactions). The **A** and **B** panels show the Similarity Network for *E. coli K12,* whereas the **C** and **D** panels indicate that for *B. subtilis 168*. E – Heatmap built from information on the relative amino acid distributions among all the MTase families detected in the Similarity Networks. *Refers to the N-terminal domain of the bi-functional MnmC enzyme.

In addition to presenting the phylogenetic occurrence of RNA MTases across bacterial species, we analyzed the sequence-based relationships among different MTase families to trace their evolutionary origin. With the aim to shed light on this topic, we performed an extensive sequence analysis using probabilistic inference methods (HMMER3 based analysis), a distant homolog searching algorithm (PSI-Coffee analysis) and the information retrieved from almost 3,000 amino acid sequences. Consequently, the respective amino acid profiles obtained for each family of RNA MTases described in *E. coli* (see Table 1) were used to rescue proteins with similar amino acid patterns along the *Escherichia coli K12* genome and other genomes from model organisms such as *Bacillus subtilis 168*. We represented the sequence matches among families (nodes) in a network fashion by scoring the interactions (edges) with a Similarity Index calculated from different alignment parameters such as length and amino acid substitutions (see Amino acid profiles at Methods). Similarity index higher than 2.5 supported trusted relationships between proteins, this is, sequence alignments at least 35 amino acids in length (~15% of the average size of MTases). Figure 3A illustrates the network representing the relationships among the different MTases in *E. coli*. Accordingly, we found RNA MTases have several sequence patterns present in other AdoMet-dependent MTases, including the Ribosome Protein Methyltransferases (orange nodes) and the MTases involved in the biosynthesis of cofactors/vitamins (gray nodes). Strikingly, no sequence-based relationships were detected with DNA MTases; these relationships would be expected given the similar nature of the substrates on which both types of MTases act. Consequently, these results indicate that the sequence similarities observed in our analysis are based on certain relationships with no bias by substrate preference. The network also represents three major lineages of MTases that are reproducible in *E. coli* and *B. subtilis* (Figure 3C), most of which are Class I MTases (a big cluster of nodes), SPOUT MTases (the gray-shaded cluster), and the RsmB/F cluster (the blue-shaded cluster). This last group of proteins was clearly separated from the other Class I MTases in *B. subtilis* (Figure 3C)*.* The clustering of the well-defined SPOUT [11,38] and RsmB/F [58] lineages also supports our results and reinforces the idea of a single lineage comprised of the majority of the Class I MTases acting in different types of substrates. After performing a cluster analysis based on edges scores and different interactions (see Amino acid profiles at Methods), the Multifunction Cluster of MTases in both model organisms was split into two sub-populations. Although no clear distribution of functions was seen, one of the groups was predominantly made up of RNA and Protein MTases, whereas the other one was constituted predominantly by the MTases involved in cofactor/vitamin biosynthesis and unknown function MTases, which could well be predicted for this molecular function.

### Origin and lineages of RNA MTases

Interestingly, a special group of MTases was always present in the transition between both the sub-populations of the Multifunction Cluster of MTases, where greater connectivity was present. Figures 3B and 3D show such relevant nodes in the network. Thus, the RsmC, PrmA, PrmB, and PrmC proteins obtained the higher connectivity values in respective networks, indicating that one of these families could be the original member of this Multifunction Cluster of MTases. These results were similarly found in the MTase networks built for *Mycobacterium tuberculosis H37Rv*, *Pseudomonas aeruginosa PA01*, *Staphylococcus aureus MRSA252* and *Thermotoga maritima* MSB8 (data not shown). A complementary analysis was performed to detect the distribution of the amino acid patterns of RNA MTases across almost 12,000 bacterial genomes in order to disclose possible founder lineages together with orthology and paralogy. In Figure 2, the distribution of the amino acid patterns is observed for the full set of RNA MTases described in *E. coli* (Table 1). Using violin plots, the large scale information obtained from the similarity networks is better shown. In global terms, the amino acid sequence patterns of RsmC and RsmD MTases are widely spread in bacterial MTases (see the above distribution; 2.5 Similarity Index). Other MTases that have the same profile are CmoA, CmoB, RsmG, RsmI and TrmN6. The amino acid patterns of these families of MTases were present in other RNA MTases and Ribosome Protein MTases. In addition to the MTase lineages observed in the similarity network analysis, this approach was useful to distinguish unique lineages constituted by RsmE, RlmM, the N-terminal domain of the MnmC, TrmD, and RlmK MTases families, thus revealing a very clear profile that supports only orthology with the highest similarity values (Similarity Index > 7.5). Interestingly, members of the SPOUT class of MTases, such as RlmB, TrmH, TrmJ and TrmL, showed a characteristic profile in terms of their amino acid sequence patterns distribution in bacterial genomes. This distribution agrees with paralogy (Similarity Index abundance between ~5 and 7), where duplication and specialization were still detectable at the sequence level [11,38]. Likewise, we detected the marked presence of RsmF paralogs, but not in RsmB. Given the poor phylogenetic distribution of RsmF (Figure 1), we hypothesized that such paralogs can perform the RsmF function; therefore, more exhaustive analyses into the phylogenetic relationship and experimental approaches to test the function of these potential new members of the cluster RsmF/B should be addressed in future studies.

### Family-specific amino acid models

Multiple sequence alignments were built for each RNA MTases family (Table 1) using iterative methods. In addition to the conserved pattern of amino acids for each RNA MTases family based on a probabilistic model for amino acid content per site (HMMER3 based analysis), we further analyzed the averaged model of amino acid content per family. We extended our study to other MTases, which are related to RNA MTases according to the similarity networks (Figures 3A-3D), to know whether RNA MTases differ from others acting on substrates other than RNA. After comparing the distribution of amino acids per family through hierarchical clustering, we observed that the entire set of RNA MTases clustered separately from those enzymes acting on non-RNA substrates (Figure 3E). We particularly aimed to disclose the specific amino acids distribution associated with the MTase function. Therefore, we split all the MTases studied into four different groups as follows: 16S MTases, 23S MTases, tRNA MTases, and non-RNA MTases; through multiple pair-wise comparisons, we detected the differential amino acid proportions among MTases for amino acids E, I, K, L, M, N, Q, R, S, and V (p < 0.016). As expected, positively charged amino acids K and R were found in a higher proportion in all the RNA MTases groups in response to the substrate they modify (p < 0.00002). Charged polar amino acids N, S and Q also showed a high distribution in all the RNA MTases groups (p < 0.016). The I, M, L, and V amino acids had a greater and significantly different distribution in all the non-RNA MTases (p < 0.001). This last observation correlated well with the substrates for these enzymes, such as proteins and coenzyme biosynthesis (biotin and ubiquinone), where hydrophobic interactions can help stabilize enzyme-substrate binding. The negatively charged amino acid such as E, but not D, was differentially found to have high proportions in all the RNA MTases groups (p < 2.0 × 10^-9^). These data were unexpected since a high density of negatively charged amino acids can repel or affect binding with a substrate to present a net electronegative charge. We hypothesized that the relevance of the presence of E in RNA MTases can be explained by counterbalancing the high proportion of positively charged amino acids in structural terms. However, we have no strong evidence to support this notion. Given the good fitted clustering of RNA MTases according to amino acid distribution, we propose that this parameter is a useful criterion to help predict bacterial RNA MTases in addition to structural and sequence evidence.

### How MTases evolve

**Figure 4:**
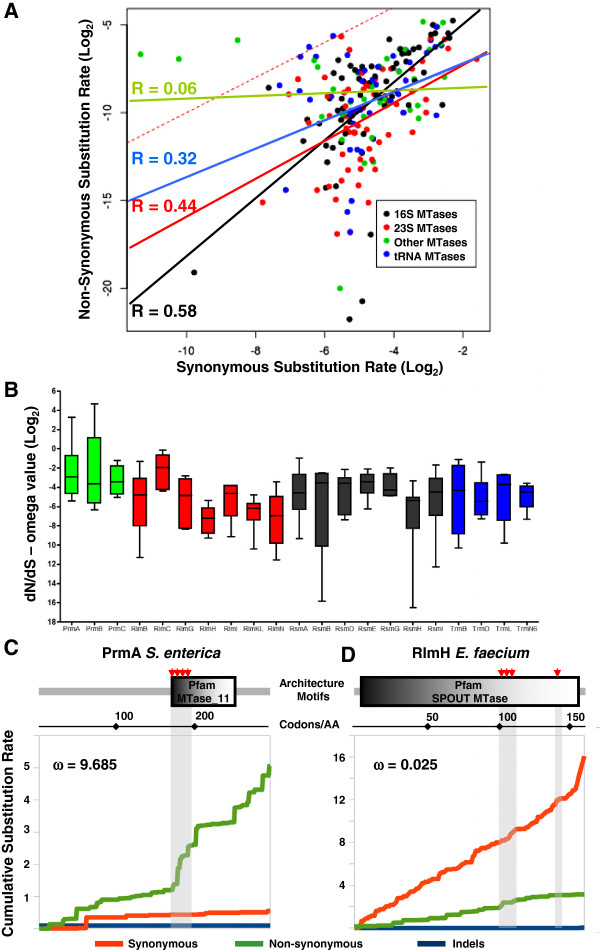
**Short-term molecular evolution of the RNA and non-RNA MTases. A** – Scatter plot showing the dN and dS Log_2_ values for each MTase studied in eight different patho-pangenomes. The MTases were classified according to subtract. The correlation coefficients for each type of MTases were calculated and plotted together with tendency lines. The red dashed line shows the neutrality boundary where the upper values are considered to be under positive selection and the lower one is considered to be under purifying (or stabilizing) selection. **B** – Boxplot showing the distribution of the (ω) omega values (Log_2_). Categorization of the MTases in agreement with the plot in panel **A**. Deep view of the synonymous and non-synonymous substitutions on *prmA* from *S. enterica***(C)***,* showing one of the highest omega values, and *rlmH* from *E. faecium***(D)***,* showing a pattern purifying selection. These plots indicate the exact sites on proteins where the synonymous and non-synonymous substitutions predominantly lie. The critical sites for protein function are highlighted in gray.

We tested all the alignments built from the RNA and non-RNA MTase families for approximately 120 empirical amino acid substitution models by using maximum likelihood approaches (see Phylogenetic analyses at Methods). After recovering the model that best explained the amino acid replacement events in each MTase family, we found that all the MTases evolved according to the LG method [82]. This indicates that MTases evolve at different rates along their sequences. This observation is consistent with the fact that most MTase families present a simple architecture consisting of a sole MTase domain. Thus, one or more functions such as substrate recognizing, specificity, cofactor binding, and catalysis (functionally and structurally compiled in a unique domain), could evolve differently than others at the MTase inside. Additonally, the model that explains evolution in MTases implies the categorization of sites according to variation level, from invariable to hypervariable sites. Categorization of site variability supports the results stated in the last section and reinforces the idea that the amino acid frequency bias is pivotal during MTases evolution and probably explains their specialization. The evolutionary pattern observed for MTases can be seen in cumulative substitution rate plot in Figure 4C. Using the pangenome information across the more than 360 genomes from the *Salmonella enterica* strains, we analyzed substitution rates for synonymous and non-synonymous amino acid replacements in one of the MTases presenting an omega value of (ω) > 1, suggesting positive selection. In the plot of accumulated substitution rates along the PrmA coding sequence, certain regions or sites where non-synonymous substitutions preferably cluster are clearly observed. This information is particularly relevant and partially explains the vast variability in MTases found as a whole.

### Selection to maintain the structure

**Figure 5:**
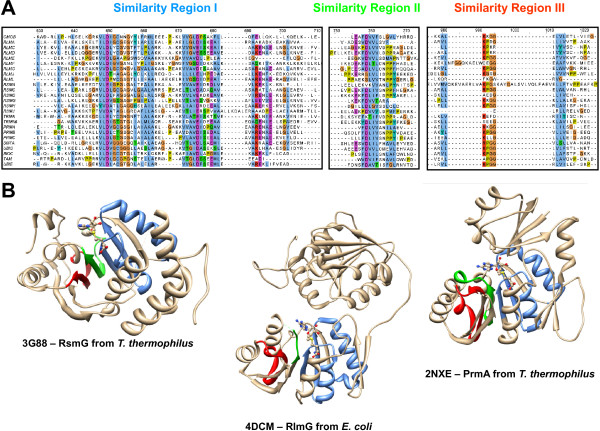
**Sequence similarity characterization among the Class I MTases.** Using the entire amino acid profiles from the set of MTases comprising the “Multifunction Cluster” in the Similarity Networks, a multiple sequence alignment was built based on the algorithms specialized in the detection of distant homologs. **A** - From the multiple sequence alignment, three different Similarity Regions, Regions I-III, with a high degree of conservation, were clearly retrieved. **B** - The three-dimensional structures depicted in the bottom panels, and regarding the different MTases families of the Multifunction Cluster, show the consensus localization of these Similarity Regions in the respective protein structures.

We performed multiple sequence alignments among all the MTases analyzed in this study using the amino acid profiles obtained through probabilistic inference and specific algorithms to detect distant homologs (PSI-Coffee based analysis). Figure 5A shows three different similarity regions in the multiple sequence alignment of several related MTases. These similarity regions are not recognized in other Class I MTases that probably conform independent lineages. Data presented in Figure 5A fit the information derived from the study of amino acid substitution model, and these regions correspond to those sites where synonymous substitutions preferentially occur. The relevance of these similarity regions was further considered from the structural point of view (Figure 5B). The similarity regions were identified and highlighted in three different types of MTases analyzed. Although the role of similarity region I is evident and has been previously seen to be involved in AdoMet binding, the role of the other two regions remains unclear. Previous analyses have linked a small motif of region II (N/D-P-P-X) with target nucleotide binding [13], but this motif is present even in some non-RNA MTases such as PrmB and PrmC. When we localized the other two similarity regions into the three-dimensional structures, we realized that they immediately lay adjacent to the first β-strand comprising the canonical AdoMet binding region (highlighted in blue). Similarity regions II and III predominantly formed the third and fourth β-strands of the characteristic β-sheet of the Rossmann Fold. Given their localization in the protein structure, they may play a critical role in structure conformation and stability where the interactions among almost all the amino acids of the similarity regions seem to be evolutionarily conserved. Additionally, we analyzed the amino acid proportions in these three similarity regions for all protein families where they were detected. We found that multiple differences in amino acid content previously detected between RNA and non-RNA MTases were abolished except for Lysine (p < 0.0156). As a consequence, this data also support the idea that Similarity Regions (I-III) evolve in the same manner in all Class I MTases probably as a consequence of their structurally role; therefore, substrate recognizing and binding roles are confined to other regions where amino acid content evolves according to target substrate.

### Short-term evolution of RNA methyltransferases (patho-pangenome genetic variability)

We studied the genetic variation of MTases in eight different human pathogens: *Acinetobacter baumannii, Staphylococcus aureus, Pseudomonas aeruginosa, Mycobacterium tuberculosis, Enterococcus faecalis, Enterococcus faecium*, *Helicobacter pylori*, and *Salmonella enterica*. After an initial examination to detect the MTases encoded in the respective genomes, coding sequences were extracted, aligned and compared in a pair-wise manner. The averaged dN (non-synonymous rate), dS (synonymous rate) and ω (dN/dS ratio) from the pair-wise comparisons were calculated and used to compare different MTases groups. The dN and dS from all the MTases gene families found in the eight patho-pangenomes are plotted in Figure 4A. The distribution of the dS and dN values was evaluated by calculating linear regression. A correlation among the members belonging to the different MTases groups was observed, and was higher in the 16S rRNA MTases genes. The tRNA and 23S rRNA MTases genes showed similar correlations, with tendency to neutrality in both cases (parallel to the dashed red line). While dN and dS values from 16S rRNA MTases seem to show a tendency to purifying or stabilizing selection, the non-RNA MTases showed a tendency to positive selection, although they presented a poor correlation coefficient, probably because of the multiple functions included in this group. We found the highest ω values in the *prmA* and *prmB* genes, whose proteins also showed higher connectivity values in the similarity networks (Figures 3A-3D). The distribution of the ω values is presented in Figure 4B for most of the recurrent MTase gene families found in the patho-pangenome analysis. This boxplot shows that *prmA*, *prmB* and *prmC,* and also *rlmC,* tended to have higher values. Conversely, the genes encoding the RNA MTases showed a distribution with lower ω values for instance those observed in *rsmB*, *rsmH*, and *rsmI*. A detailed view of an MTase evolving under clear positive selection and another one evolving under purifying selection is provided in Figures 4C and 4D, respectively. Non-synonymous substitutions found in the *prmA* gene from *S. enterica* are confined to the amino acid region belonging to the AdoMet binding motif or Similarity Region I (see Figure 5A). In contrast, the MTases genes under the purifying selection (Figure 4D) presented a similar synonymous substitution rate along the gene with no particular concentration of non-synonymous substitutions in any region of the protein.

### Genetic variability in antibiotic resistance-associated RNA MTases

The rRNA MTases, especially those acting on 23S rRNA, showed the lowest dN values, indicating strong purifying selection (Figure 4A) in human pathogens. As a consequence, we wanted to further explore the cumulative dN rate in some of these genes with the aim to retrieve evolutionary information from patho-pangenome structure and its possible predisposition to acquire antibiotic resistance. The 16S rRNA MTase RsmG and 23S rRNA MTase RlmN are associated with antibiotic resistance in a wide variety of bacteria, and some of them recurrent human pathogens [25-27,77,78,83]. After analyzing the pattern of the cumulative dS and dN rates along the *rsmG* and *rlmN* genes, we found that ω values were higher in *rsmG* than in *rlmN* in all the pangenomes analyzed (Additional file 1: Figure S1). This difference was most obvious in *E. faecalis,* where *rlmN* had almost null non-synonymous substitutions (>300-fold). The cumulative dS rate pattern was similar in both, indicating that synonymous substitutions occur at the same rate along the respective genomes. When codon hotspot sites for protein inactivation are taken into account [27,84-87], as well as Similarity Regions among Class I MTases described here (Figure 5A), difference among the cumulative dN rates between *rlmN* and *rsmG* indicates that this last could undergo a selection which would affect pivotal sites for protein function, essentially where AdoMet binding underlies. A site-by-site analysis of cumulative synonymous and non-synonymous substitutions in the *rsmG* AdoMet binding site showed that non-synonymous substitutions fall outside critical sites for protein function (Additional file 2: Figure S2).

## Conclusions

The study of RNA MTases can help to understand their role in translation. Given the enormous variability among the RNA MTases, their evolutionary relationship is unclear. Here we presented data to support the notion that several MTases emerge from one common ancestor. Nevertheless, we could not identify the ancestral sequence. We reviewed the entire set of RNA MTases described for *Escherichia coli,* and we disclosed a core set of RNA MTases in Eubacteria by studying phylogenetic profiles in different phyla. We identified approximately 13 RNA MTase families that are highly conserved across bacterial species which probably represent the core of methylations for the proper function of tRNA and rRNA. From the amino acid and DNA sequences analyses, we showed that most Class I RNA MTases are related to Ribosomal Protein MTases, such as PrmA, PrmB, and PrmC, as well as other MTases that act in cofactor/vitamin biosynthesis. The Prm proteins show many links with RNA MTases (Figure 3) and their high proportion of non-synonymous substitutions could support their role as a founder lineage of Class I MTases included in the “Multifunction Cluster” defined here. We could identify unique lineages through massive sequence comparisons using the genomic information of almost 12,000 bacterial genomes. The RNA MTases that seem to be unique in sequence terms are RsmE, RlmK, TrmD, RlmM, RlmN and the N-terminal domain of the bi-functional MnmC MTase. These families, together with the three different clusters evidenced by our similarity network analysis, indicate that RNA MTases diversity can be explained, at least from the emergence of nine MTase lineages. Although we have not taken into account other important groups of enzymes, such as DNA MTases, our data indicates that multiple emergence events explain the vast diversity of MTases. We also found that despite the sequence relationships, RNA MTases, and those acting in different molecules; diverge in the amino acid content, a fact that well matches the function associated with different MTases. Members of the “Multifunction Cluster” present three clear similarity regions (Figure 5). By combining the intensive amino acid sequence, the evolutionary model prediction and the molecular evolution analyses provided evidence supporting the idea that AdoMet-dependent Class I MTases are under strong purifying selection to retain the protein structure and cofactor binding site. We present a patho-pangenome molecular evolution analysis to define the short-term evolution pattern of a large set of RNA MTases and non-RNA MTases for the purpose of linking their evolution with pathogenesis. The acquisition and development of antibiotic resistance is a common feature among persistent infections. This has been strongly linked to some methylations in rRNA [27,77,78], and the mechanisms for progression from low level resistance to a high level is still unclear [27,28]. We found that rRNA MTAses evolve close to neutrality with very low non-synonymous substitution rates. We found that human pathogens are prone to accumulate non-synonymous substitutions outside critical sites of RNA MTases (Additional file 2: Figure S2). Based on these data, RNA MTases in human pathogens seem to follow patterns of evolution observed for MTases. This pattern is widespread among MTases and even in those associated to mutation-dependent mechanism to acquire and develop antibiotic resistance. Data obtained from different approaches used in this study fit well patterns of variation observed for bacterial AdoMet-dependent non-coding RNA MTases, and they may represent a response to substrate specialization but retaining ancient functional modules.

## Methods

### Sequence analyses

The phylogenetic distribution and relationships of RNA MTases were studied by downloading a set of more than 3,000 protein sequences grouped into 34 families based on the full core of RNA MTases functionally characterized to act in *E. coli* rRNAs and tRNAs [7,9,18,27,34,39-69] (Table 1). Using the amino acid sequences of *E. coli* RNA MTases as queries [88], a Blastp search against the non redundant Reference Sequences Database at NCBI [89] was conducted with default parameters (http://blast.ncbi.nlm.nih.gov/Blast.cgi) [90]. We explored the phylogenetic distribution of the RNA MTases homologs in major bacteria groups (i.e., Acidobacteria, Actinobacteria, Bacteroidetes, Chloroflexi, Chlamydiae, Cyanobacteria, Deinococci, Firmicutes, Fusobacteria, Proteobacteria, Spirochaetes, Tenericutes, and Thermotogae). Based on pair-wise comparisons with an alignment coverage of >75% and an alignment score of >60 bits, we retrieved more than 3,000 different sequences representative of the diversity of RNA MTases for each bacterial phylum. Each family of RNA MTases was then aligned using the Probcons software, v1.12, with 1,000 passes of iterative refinement [91], followed by filtering for gaps.

### Amino acid profiles

High quality alignments were used to built respective amino acid profiles were constructed using the HMMER3 algorithm and default parameters [92]. The protein architecture was examined using the respective HMM-based amino acid profiles and the SMART server [93]. The averaged amino acid distribution per family was analyzed using hierarchical clustering. Consequently, the heatmaps of amino acid composition were generated using the gplots library in R [94] with previous log2-transformation of frequencies and clustering with a complete method and euclidean distance. The RNA MTase networks, based on probabilistic inference methods and sequence relationships among RNA MTases, were constructed for model organisms, such as *Escherichia coli K12* (GenBank id, NC_000913) and *Bacillus subtilis 168* (NC_00949), using Biolayout Express 3D and the Markov Clustering Algorithm (MCL) [95]. The clustering of nodes was performed for *Mycobacterium tuberculosis H37Rv* (NC_018143), *Pseudomonas aeruginosa PA01* (NC_002516), *Staphylococcus aureus MRSA252* (NC_002952), *Thermotoga maritima* MSB8 (NC_021214). As a result, the amino acids profiles based on Hidden Markov Models (HMM) for the 34 RNA MTases families and the nine additional families of *E. coli* non-RNA MTases (BioB, BioC, PrmA, PrmB, PrmC, SmtA, Tam, UbiE, and UbiG) were compiled and indexed in an HMM database using the *hmmpress* algorithm contained in the HMMER3 package. A search for the proteins related to the MTases proteins was done using the *hmmscan* algorithm (HMMER3 package) with a threshold score of >25. Proteins sharing a sequence similarity against the MTase profiles compiled in the HMM database were ranked according to a normalized Similarity Index = Log_2_ [(Lt/Lp) × S], where Lt is equal to the length of the sequence aligned in the target, Lp is the total length of the query amino acid profile, and S is the alignment score. This Similarity Index was used as a measurement of the sequence relationships among the MTases reflecting the edges in the protein networks.

### Phylogenetic analyses

Relationships among the RNA MTAses were analyzed by two approaches. First, occurrence probabilities for all the amino acids in each MTase family. The HMM profiles built with the HMMER3 software. All probabilities were set as variables in a similarity matrix, and a dendrogram was constructed using the UPGMA algorithm with Pearson’s coefficient and 100 bootstrap replicates on the following web server: http://genomes.urv.cat/UPGMA/index.php[96]. The multiple pair-wise comparisons made among the RNA MTase groups were calculated in R v3.0 (http://www.r-project.org/) and an ANOVA test with Bonferroni correction was used. The second approach performed to disclose the evolutionary model for each RNA MTase family analyzed. Likelihoods for 120 empirical models (containing 15 different matrices) implemented in ProtTest v3.3 were calculated [97-99]. The best model was selected according to the smallest corrected Akaike Information Criterion (AICc). The Similarity Regions among MTases was obtained by the multiple sequence alignment of the respective amino acid profiles obtained from HMMER3 and using iterative algorithms for distantly related sequences (PSI –Coffee at T-Coffee web server, http://www.tcoffee.org) [100,101].

### Genome-scale analysis of bacterial pathogens

Presence of different RNA MTases and related proteins was massively detected in almost 12,000 fully-sequenced bacterial genomes publicly available in the Pathosystems Resource Integration Center (PATRIC). Approximately 50 million encoded proteins were tested to match the RNA MTases using probabilistic inference methods, as previously stated. The alignment hits and respective Similarity Index were clustered according to RNA MTase similarity. Then the violin density plots were drawn in R v3.0 (http://www.r-project.org/) and the ggplot2 package [102]. According to the Similarity Index distribution among the different protein families, the hits showing a Similarity Index higher than 7.5 were selected as true orthologs, whereas those hits showing a Similarity Index lower than 5 and higher than 2.5 were considered to be proteins that were phylogenetically related to the RNA MTases, based on the criteria of at least 35 aa in length and a score ~30. The alignment hits showing a Similarity Index higher than 5 were and lower than 7 were selected as the potential paralogs. These latter proteins, which exhibited a potential paralogy with certain RNA MTases, were extracted and functional prediction was assessed according to the sequence, motifs, and architecture criteria.

### Genetic variability in patho-pangenomes

The intra-species molecular evolution of the RNA MTases in human pathogens was investigated by analyzing the genetic variability in these genes in almost 2,000 genomes. Consequently, the coding sequences for all the RNA MTases studied, when presented, were extracted from pangenomes from eight common human pathogens: *Acinetobacter baumannii* (186 genomes)*, Staphylococcus aureus* (438 genomes)*, Pseudomonas aeruginosa* (47 genomes)*, Mycobacterium tuberculosis* (75 genomes)*, Enterococcus faecalis* (271 genomes)*, Enterococcus faecium* (229 genomes), *Helicobacter pylori* (243 genomes) and *Salmonella enterica* (393 genomes). They were respectively aligned using iterative and accurate methods [103,104]. The synonymous and non-synonymous substitution rates were calculated in a pair-wise fashion using SNAP calculator v1.1 [105] and by correcting transitional substitutions [106]. As a result, the synonymous and non-synonymous substitution rates and the proportions for the transitional substitutions were obtained and used for the comparisons made among the MTases families. Linear regression and multiple pair-wise comparisons were done among the RNA MTase groups, and were calculated in R v3.0 (http://www.r-project.org/) using an ANOVA test with Bonferroni correction.

## Competing interests

The authors declare that they have no competing interests.

## Author’s contribution

ABP designed this study. JMR and SCB carried out the sequence and phylogenetic analyses. MC and JDP assisted sequence and phylogenetic analyses and computing performance. ABP and JMR worked in manuscript preparation. All authors read and approved the final manuscript.

## Supplementary Material

Additional file 1: Figure S1 The Cumulative Substitution Rate plots for genes *rlmN and rsmG*. A comparative analysis done with the genes and pathogens for the distribution of the synonymous and non-synonymous substitutions in genes *rlmN* and *rsmG* is shown. Those genes are well known to be associated to antibiotic resistance. Critical sites for the protein function are highlighted in gray. The correlation between the high accumulation of the non-synonymous substitutions and hotspots for the functional inactivation of RsmG are more clearly inferred. Click here for file

Additional file 2: Figure S2 Close view for Cumulative Substitution Rate in *rsmG.* Two plots showing the distribution of the synonymous and non-synonymous substitutions at amino acid sequence level in *rsmG* genes from *H. pylori* and *P. aeruginosa*. Red lines show cumulative synonymous substitutions and green lines show non-synonymous substitutions. Hotspots for protein inactivation (gray shaded amino acid positions) were compiled from [27,85]. Click here for file
