# Supplementary figures and images for: Evolutionary and sequence-based relationships in bacterial AdoMet-dependent non-coding RNA methyltransferases
Source: BMC Res Notes. 2014 Jul 10;7:440. doi: 10.1186/1756-0500-7-440 (PMC4119055; doi:10.1186/1756-0500-7-440)

Cumulative Substitution Rates

*rlmN*

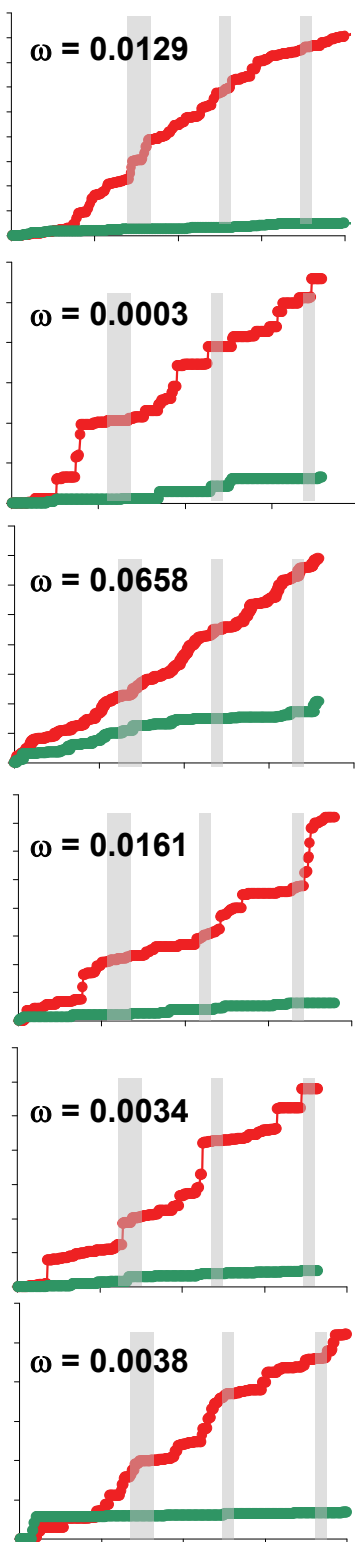

*rsmG*

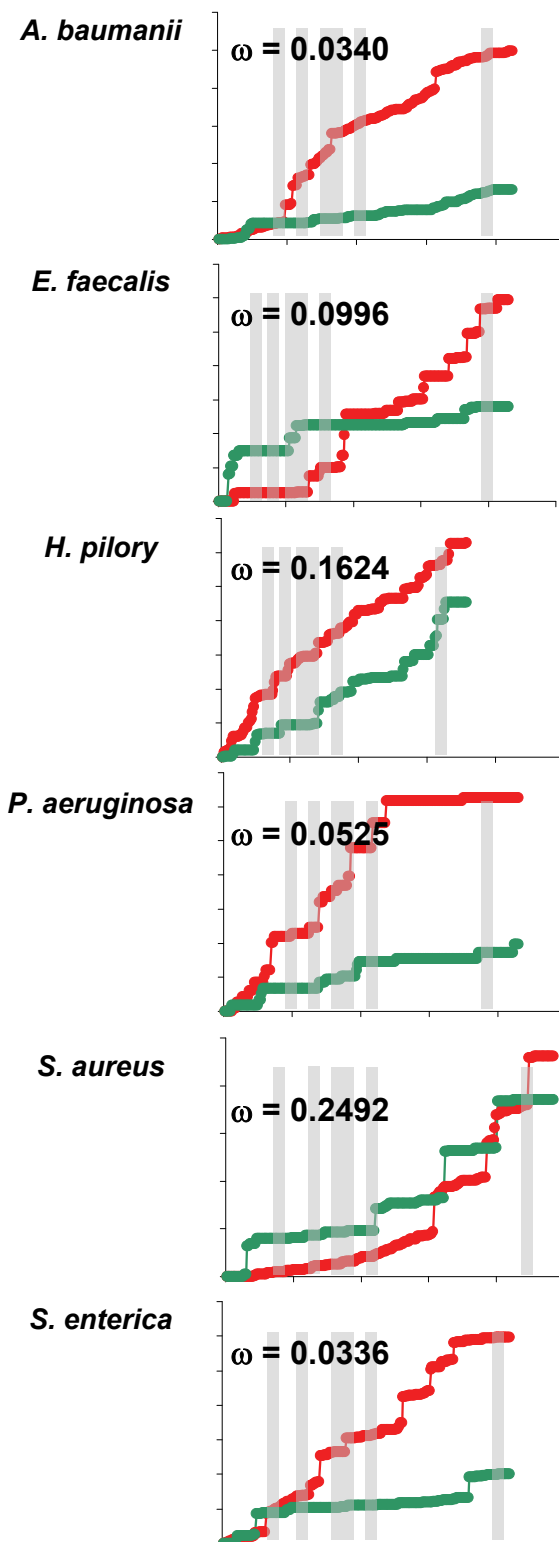

— Synonymous  
— Non-synonymous

Supplement: Additional file 1: Figure S1 — The Cumulative Substitution Rate plots for genes rlmN and rsmG. A comparative analysis done with the genes and pathogens for the distribution of the synonymous and non-synonymous substitutions in genes rlmN and rsmG is shown. Those genes are well known to be associated to antibiotic resistance. Critical sites for the protein function are highlighted in gray. The correlation between the high accumulation of the non-synonymous substitutions and hotspots for the functional inactivation of RsmG are more clearly inferred. [file 1756-0500-7-440-S1.pdf]

Cumulative Substitution Rates

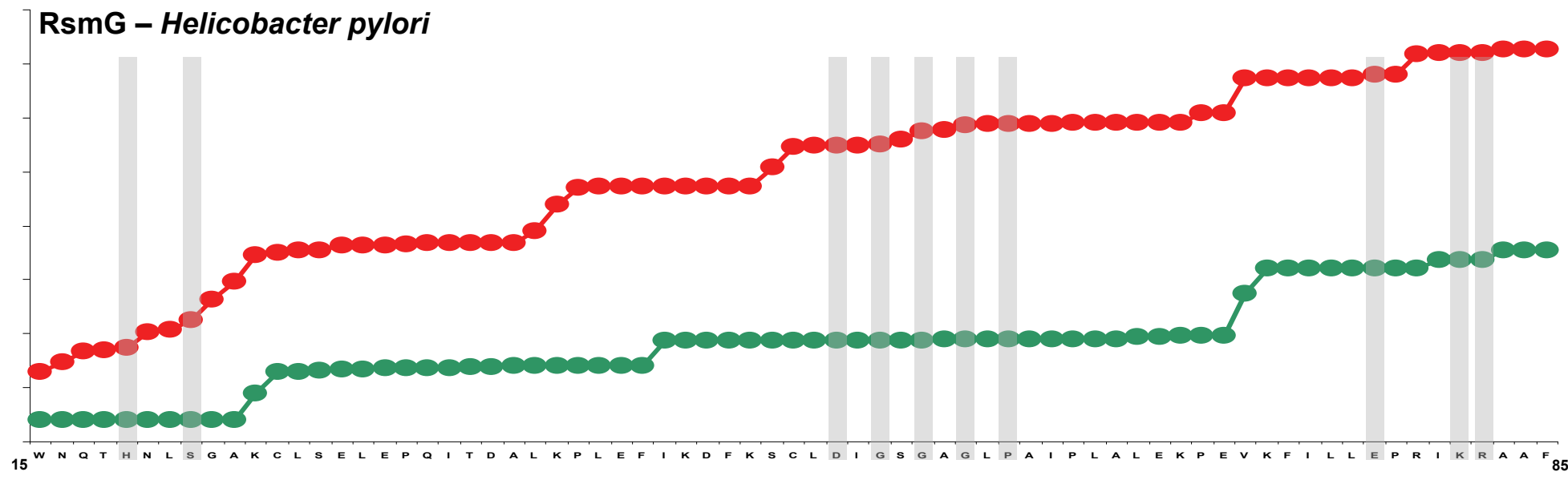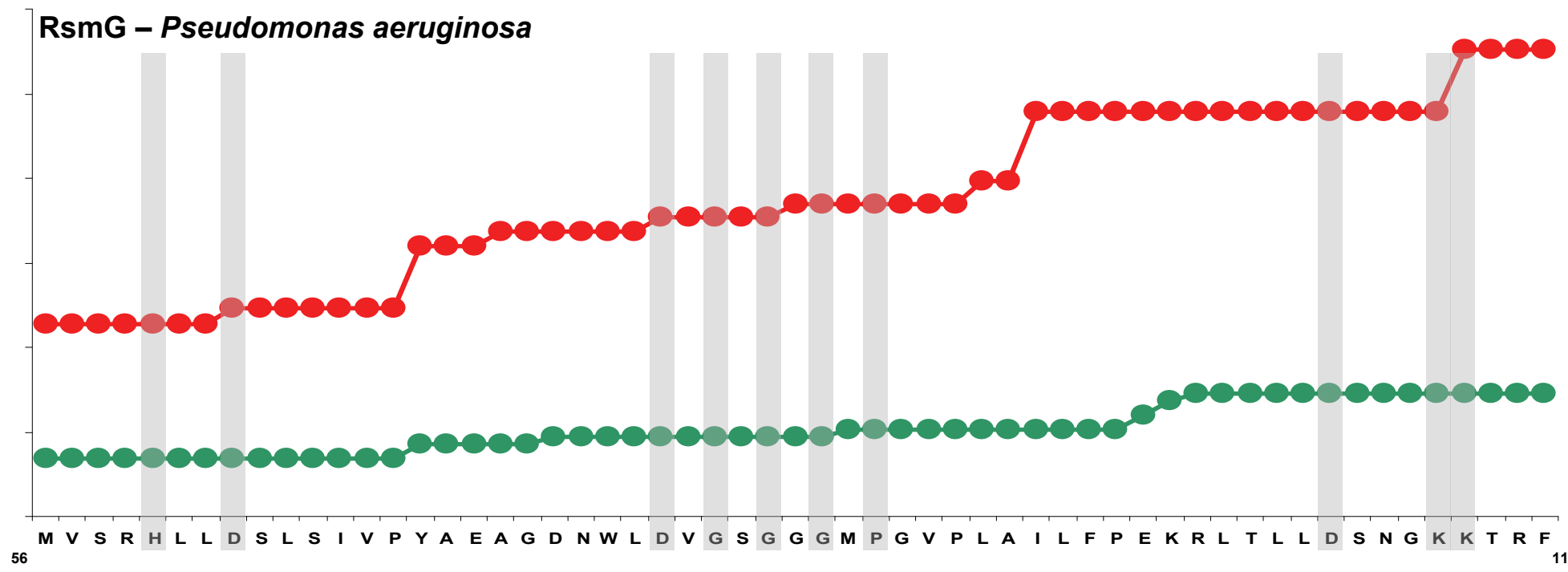

Supplement: Additional file 2: Figure S2 — Close view for Cumulative Substitution Rate in rsmG. Two plots showing the distribution of the synonymous and non-synonymous substitutions at amino acid sequence level in rsmG genes from H. pylori and P. aeruginosa. Red lines show cumulative synonymous substitutions and green lines show non-synonymous substitutions. Hotspots for protein inactivation (gray shaded amino acid positions) were compiled from [27,85]. [file 1756-0500-7-440-S2.pdf]
